# Letter to the editor regarding the case report: unregulated medication use and complications: is self-medication a common problem with anti-tuberculous therapy (ATT)? Role for patient education

**DOI:** 10.1186/s12879-024-09170-x

**Published:** 2024-03-04

**Authors:** Vijay Alexander, Jasper Rathinam J., John Titus George, Jackwin Sam Paul

**Affiliations:** 1 Department of Hepatology, Christian Medical College, 632002 Vellore, India; 2 Division of Critical Care, Christian Medical College, Vellore, India; 3 Department of Gastroenterology, Christian Medical College, Vellore, India; 4 Department of Community Health, Christian Medical College, Vellore, India

**Keywords:** Tuberculosis, self-medication, Post-tuberculosis sequelae, Patient education, Anti tuberculous therap, ATT

To the Editor,

We read with great interest the case report titled ‘unregulated medication use and complications: a case study of prolonged self-treated tuberculosis in Nepal’ [1]. The authors describe a middle-aged gentleman diagnosed with tuberculosis once, later continues to self-medicate for years without seeking medical attention.

The case in discussion is unusual for many reasons, and raises multiple questions relevant to the management of tuberculosis. While poor adherence to the prescribed course of anti-tuberculous therapy and defaulting treatment are commonly reported, self-medication and prolonged intake over and above the prescribed duration is extremely uncommon [2]. Studies have assessed multiple interventions such as direct observed therapy administered by trained health workers, SMS combined with education, drug reminder box and reinforced counselling method to improve medication adherence to ATT [3]. Since the problem identified is poor adherence, these interventions target those that require ATT. Most health programmes do not have long follow up care of patients cured of TB. As stated above, most studies do not report self-treatment with ATT as a problem among TB patients beyond prescribed course of treatment.

Over the counter medication use and self-medication is a common problem in low- and middle-income countries where there are few safety checks in place to limit the dispensing of medications without prescription. Commonly bought medications include antipyretics, antibiotics, analgesics, and steroids [4]. However, over-medication with ATT is rare and has not been previously reported in literature. Common reasons for self-medication of various drugs include urgency of the situation, financial constraints, previous experience, advice obtained from paramedics and pharmacists, easy availability of drugs, non-availability of medical insurance to make a visit to the clinic, lack of time and poor access to a health care facility [5].

This case highlights the importance of patient education in tuberculosis, especially regarding the duration of therapy, documentation of sputum conversion and microbiological cure and, possible long-term sequelae of tuberculosis infection. A brief note on the patient’s perspective would have been extremely beneficial and would have helped the authors and the readers understand his concerns and thought process behind treating himself with ATT for years together. We can only speculate the reasons why this patient resorted to prolonged period of self-medication with ATT. It is possible that he was not cognizant of the fact that tuberculosis is curable with a finite course of appropriate ATT. He may also have been unaware of sequelae of past tuberculosis such as bronchiectasis and lung fibrosis. The stigma of living with tuberculosis may also have been a motivating factor to take treatment plan into his own hands without involving family or healthcare professionals.

This lays the foundation for healthcare system research, with a crucial focus on identifying the tipping point within the system. Employing a mixed-methods approach, we can quantify the myriad challenges patients encounter after receiving ATT but also gain insights into the factors that either support or hinder their access to proper follow-up care. This would entail an initial qualitative data collection through in-depth interviews and focus group discussions (FGDs) to delve into the reasons for non-adherence to anti-tuberculous treatment done in the exploratory phase. Following this, a quantitative phase should be performed by designing a cross-sectional study based on identified qualitative themes, aiming to quantify specific factors related to non-adherence. Subsequent research would triangulate by aligning qualitative and quantitative findings to reveal convergent or divergent patterns. These insights will not only inform public health system improvements but also assist clinicians in crafting more appropriate management and patient education strategies.

## Data Availability

Not applicable.

## Abbreviations

ATT: Anti–tuberculous therapy
TB: Tuberculosis
SMS: Short Message Service
FGD: Focus Group Discussion
